# Two-year Opioid Prescription Trends in Local Sanitary Agency Naples 3 South, Campania Region, Italy. Descriptive Analyses and AI-based Translational Perspectives

**DOI:** 10.37825/2239-9747.1047

**Published:** 2024-02-27

**Authors:** Marco Cascella, Maurizio Capuozzo, Francesco Ferrara, Alessandro Ottaiano, Francesco Perri, Francesco Sabbatino, Valeria Conti, Vittorio Santoriello, Alfonso Maria Ponsiglione, Maria Romano, Francesco Amato, Ornella Piazza

**Affiliations:** a Department of Medicine, Surgery and Dentistry, University of Salerno, Baronissi, 84081, Salerno, Italy; b Pharmaceutical Department, ASL Napoli 3 Sud, Ercolano, 80056, Naples, Italy; c Istituto Nazionale Tumori di Napoli, IRCCS “G. Pascale”, via M. Semmola, 80131, Naples, Italy; d Department of Information Technology and Electrical Engineering, University of Naples Federico II, 80125, Naples, Italy

**Keywords:** Opioids, Cancer pain, Opioid crisis, Non-cancer pain, Artificial intelligence, Rapid onset opioids, Fentanyl, Tapentadol, Buprenorphine, Fentanyl pectin nasal spray, Defined daily dose

## Abstract

**Aims:**

This study delves into the two-year opioid prescription trends in the Local Sanitary Agency Naples 3 South, Campania Region, Italy. The research aims to elucidate prescribing patterns, demographics, and dosage categories within a population representing 1.7% of the national total. Perspectives on artificial intelligence research are discussed.

**Methods:**

From the original dataset, spanning from January 2022 to October 2023, we processed multiple variables including demographic data, medications, dosages, drug consumption, and administration routes. The dispensing quantity was calculated as defined daily doses (DDD).

**Results:**

The analysis reveals a conservative approach to opioid therapy. In subjects under the age of 20, prescriptions accounted for 2.1% in 2022 and declined to 1.4% in 2023. The drug combination paracetamol/codeine was the most frequently prescribed, followed by tapentadol. Approximately two-thirds of the consumption pertains to oral formulations. Transdermal formulations were 15% (fentanyl 9.8%, buprenorphine 5.1%) in 2022; and 16.6% (fentanyl 10%, buprenorphine 6.6%) in 2023. These data were confirmed by the DDD analysis. The trend analysis demonstrated a significant reduction ( *p* < 0.001) in the number of prescribed opioids from 2022 to 2023 in adults (40–69 years). The study of rapid-onset opioids (ROOs), drugs specifically used for breakthrough cancer pain, showed higher dosage (>267 mcg) consumption among women, whereas a lower dosage (<133 mcg) was calculated for men. Fentanyl pectin nasal spray accounted for approximately one-fifth of all ROOs.

**Conclusion:**

Despite limitations, the study provides valuable insights into prescribing practices involving an important study population. The findings underscore the need for tailored approaches to prescribing practices, recognizing the complexities of pain management in different contexts. This research can contribute to the ongoing discourse on opioid use, advocating for innovative strategies that optimize therapeutic outcomes while mitigating potential risks.

## 1. Introduction

The use of opioids remains a cornerstone in the treatment of cancer-related pain [1]. These medications play a pivotal role in alleviating pain and enhancing the quality of life for individuals battling cancer [2]. On the other hand, the application of opioids in the management of chronic non-cancer pain remains a contentious subject, with divergent perspectives on their efficacy, safety, and long-term implications [3].

The persistent and multifaceted challenges associated with opioid use have tossed the issue into the forefront of global public health debate [4]. The opioid crisis, marked by the alarming surge in opioid-related morbidity and mortality, has emerged as a critical concern demanding urgent attention and innovative solutions [5]. The phenomenon represents an unprecedented confluence of factors, including overprescription, illicit drug use, and the wide diffusion of potent synthetic opioids [6]. Significantly, this crisis has led to a surge in opioid-related overdoses and deaths, posing a substantial challenge to healthcare systems worldwide [7].

The inadequate appropriateness in prescribing opioids is a key factor contributing significantly to the unfolding dynamics of the opioid crisis [8]. Consequently, the assessment of opioid prescribing patterns can represent an effective strategy to identify potential inaccuracies and prompt appropriate corrective measures [9,10]. In the United States, for example, addressing and strengthening state prescription drug monitoring programs (PDMPs) is a commonly adopted policy measure in response to the opioid epidemic [11].

In this complex scenario, information regarding prescription methods, types of molecules, overall consumption, dosages, prescribed formulations, the use of drugs specifically approved for cancer pain, and variations based on demographic factors can serve as a crucial study resource, particularly when conducting a trend analysis.

Based on these premises, we conducted an analysis of opioid prescriptions in a large population sample. By scrutinizing prescribing patterns in this expansive cohort, our study aimed to shed light on the intricacies of opioid management, providing valuable insights into the diverse factors influencing prescription practices and paving the way for informed strategies to optimize therapeutic outcomes while mitigating potential risks.

## 2. Methods

### 2.1. Study population

**Fig. 1:**
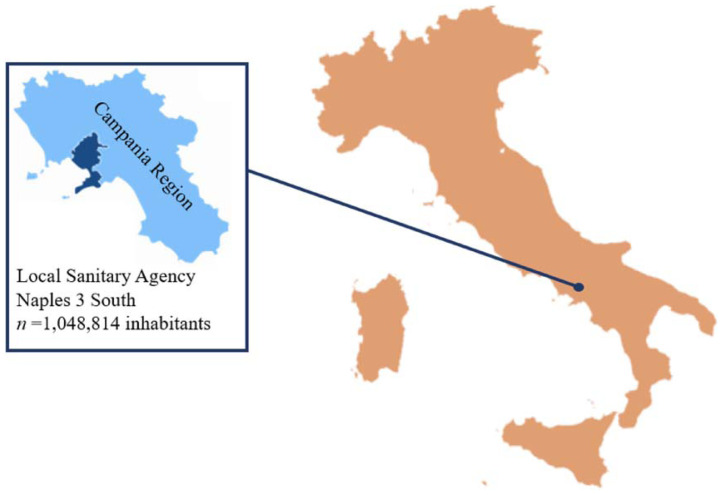
The Local Sanitary Agency Naples 3 South within the Campania region (Italy).

We analyzed prescribing patterns associated with the Local Sanitary Agency (LSA) Naples 3, in South Italy (i.e., LSA, NA 3 South). According to the Italian National Institute of Statistics (ISTAT), the reference population consists of 1,048,814 inhabitants [12] (Fig. 1).

### 2.2. Data mining and processing

The dataset comprising all opioid prescriptions from January 2022 to October 2023 in the Local Sanitary Agency Naples 3 South (Campania Region, Italy), was used to identify opioid prescriptions. The prescription dataset was divided into records that aggregate opioid prescriptions based on age groups (every 5 years), gender, and dosage. The prescription data was categorized to group opioid prescriptions by age (in 5-year intervals), gender, and dosage. To analyze trends and variations across two consecutive years, we separated the dataset into two distinct periods: January to December 2022 and January to October 2023. In these datasets, the variables extracted for the analyses included gender, age, medications, the number of prescriptions, and the sum of drug units considered as the total quantity of prescribed drugs. Drugs were categorized based on the Anatomical Therapeutic Chemical (ATC) classification system [13]. The analysis also encompasses non-opioid medications like non-steroidal anti-inflammatory drugs (NSAIDs) and paracetamol when they are combined with opioids. Additionally, in Italy, the weak opioid codeine is marketed exclusively in combination (with NSAIDs or paracetamol).

To enhance data processing, the variable age was segmented into nine groups, each spanning a range of ten years. To assess drug consumption, the totals of prescribed drugs were computed according to medications and drug packages used. Therefore, we have grouped all formulations of a type of opioid, distinguishing dosages, and administration routes. This strategy allowed for further analysis focused on the quantity of prescribed drugs and dosages. The dispensing quantity was calculated in defined daily doses (DDD). It is a standardized unit used to quantify drug consumption, representing the average daily amount of a drug prescribed for the treatment of a specific condition [14].

The analyses included the route of opioid administration and opioid prescriptions based on age. More precisely, the study defined individuals under the age of 39 as young, those within the age range of 40**–**69 as adults, and individuals aged 70 and above as elderly [15]. Finally, we investigated differences in opioid prescriptions between 2022 and 2023 and performed the assessment of the rapid-onset fentanyl (ROO) prescription across various genders and age groups. This category includes oral transmucosal fentanyl citrate (OTFC), fentanyl buccal tablet (FBT), fentanyl buccal soluble film (FBSF), sublingual fentanyl (SLF), and fentanyl pectin nasal spray (FPNS). To group the various ROO formulations, we classified the dosages into three categories: low (<133 mcg), medium (133**–**267 mcg), and high dosages (>267 mcg).

The datasets used and analyzed for the current investigation are available from the corresponding author upon reasonable request.

### 2.3. Statistics

The Excel Data Analysis ToolPak, IBM SPSS Statistics (V 29.0.1.0), and Matlab R2023B were implemented for statistical analysis. Utilizing SPSS software, an assessment of data normality was conducted using the Shapiro-Wilk test for each age group. Upon discovering non-normal distributions across groups, the nonparametric Kruskall**–**Wallis test was employed.

The Dunn**–**Bonferroni test was utilized to identify significant results among the pairs of groups analyzed, with consideration given to adjusting the *p*-values using the Bonferroni error correction method [16]. The Mann-Whitney test was conducted using the Statistics and Machine Learning Toolbox in Matlab to analyze the significance of specific pairs. The comparison of age groups between the two datasets was performed using the Mann-Whitney test, employing the previously described methods.

## 3. Results

Out of the original data of 3223 records of the 2022 dataset, 16 records were excluded from the study due to missing gender and age information. Therefore, 3207 records were included in the final analysis. The second dataset encompassed all records collected between January and October 2023. In this dataset, out of the original 3223 records, 26 were excluded for missing data and, finally, 3207 records were further analyzed.

### 3.1. Gender distribution

**Fig. 2:**
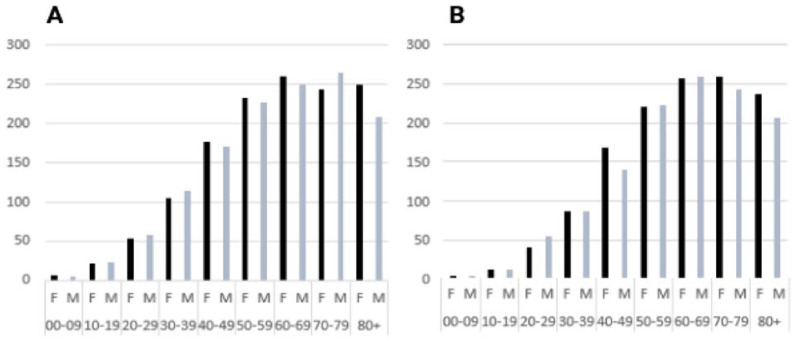
Age and gender distribution. January**–**December 2022 (n = 2671; 532 missing data) (A); January**–**October 2023 (n = 2671; 498 missing data) (B).

Figure 2 illustrates the gender distribution across nine age classes: 00**–**09, 10**–**19, 20**–**29, 30**–**39, 40**–**49, 50**–**59, 60**–**69, 70**–**79, 80+. In the first-year dataset (i.e., 2022), there were prescriptions for 1322 male and 1349 female individuals (Fig. 2A). For the second dataset (i.e., 2023), records from 1238 male and 1354 female subjects were collected (Fig. 2B). For individuals aged below 20, the prescription rates were 2.1% in 2022 and decreased to 1.4% in 2023.

**Fig. 3:**
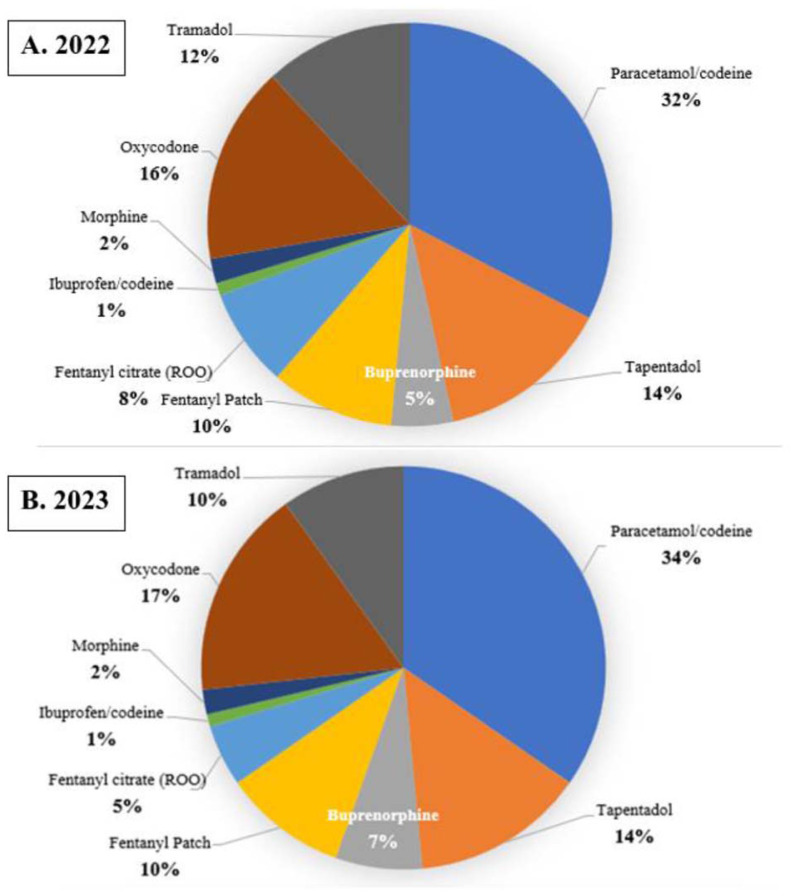
Drug prescriptions in 2022 (A) and 2023 (B). The label “Buprenorphine” refers to the patch formulations. Fentanyl citrate is the rapid-onset opioids (ROO) category. The category “Morphine” includes morphine hydrochloride and morphine sulfate. Oxycodone includes the combination of oxycodone and paracetamol (5% and 6%, in 2022 and 2023 respectively), and oxycodone plus naloxone (10% in both years). Other opioids such as hydromorphone accounted for <0.1% in both years.

The aggregate sum of drugs prescribed is shown in Fig. 3. The drug combination paracetamol/codeine was the most frequently prescribed drug in both datasets, accounting for 32% in 2022, and 34% in 2023, respectively. Considering weak opioids (codeine and tramadol), we calculated 44.3% in 2023, and 45% in 2023. Tapentadol was the second most prescribed opioid (14% in both considered years). Transdermal formulations were 15% (fentanyl 9.8%, buprenorphine 5.1%) in 2022; 16.6% (fentanyl 10%, buprenorphine 6.6%) in 2023. Among drugs specifically indicated for cancer pain, fentanyl citrate (ROOs category) accounted for 7.7%, in 2022 and 4.8%, in 2023. Within the group of ROOs, FPNS comprised 19% in 2022 and 17% in 2023, respectively.

**Fig. 4:**
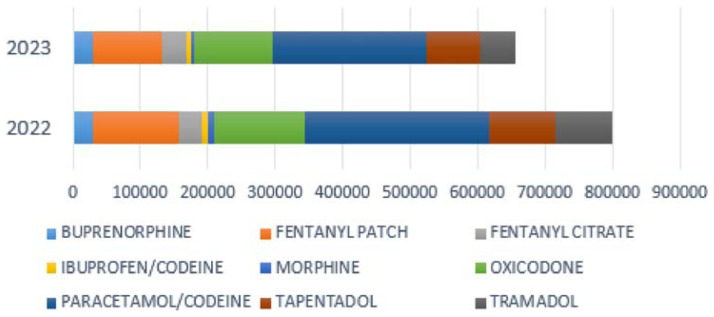
The dispensing quantity calculated as defined daily doses (DDD). From 2022 to 2023, there has been an overall reduction in DDD of approximately 18%.

These findings were verified through the analysis of DDD (Fig. 4).

To assess drug consumption, the cumulative use of medications was calculated within the different age classes. The cumulative use refers to the number of prescriptions and drug packages for each medication (Table 1).

Concerning the cumulative use of opioids for age, the statistical analysis demonstrated a non-normal distribution of the data within each age group across all datasets, as indicated by the Shapiro-Wilk test. When assessing variations among age categories in each dataset, the Kruskal**–**Wallis test identified a significant difference within the 2022 dataset ( *p* = 0.006).

The Dunn**–**Bonferroni test revealed a significant difference specifically within the Adult and Elderly categories for 2022, confirming this result after applying the Bonferroni correction for multiple tests. This aimed to identify pairs exhibiting notable differences (Table 2).

**Fig. 5:**
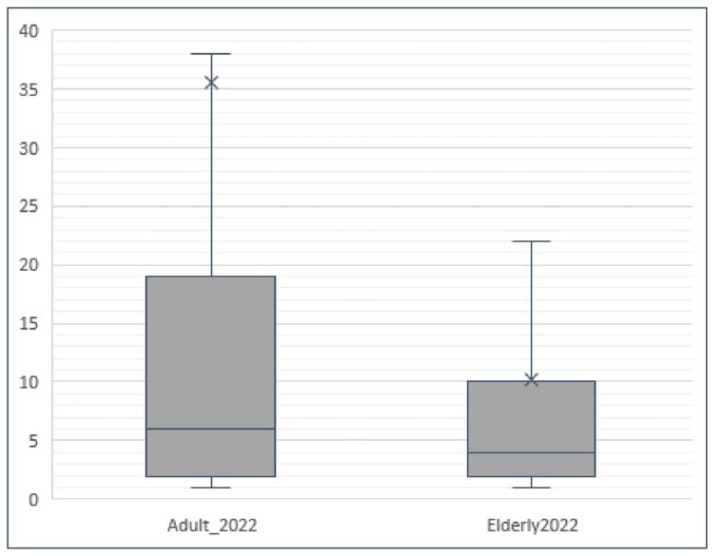
Boxplot of drug consumption in terms of pieces prescribed for adults and elderly people from January to December 2022. ( p < 0.001). Adult = 40**–**69 years; Elderly >70 years.

The result was confirmed by the Mann-Whitney test, wherein the obtained p-value was less than 0.001 ( *p* < 0.001), indicating a highly significant difference (Fig. 5).

Regarding the second dataset encompassing patients considered from December to October 2023, the was no significant difference between at least one pair of groups ( *p* = 0.161). It was confirmed by the Dunn**–**Bonferroni pairwise test (Table 3).

Trend analysis. When examining differences in drug consumption between 2022 and 2023 for each age group, the data exhibited a non-normal distribution. Consequently, the Mann-Whitney pairwise test was conducted to assess the variability (Table 4).

**Fig. 6:**
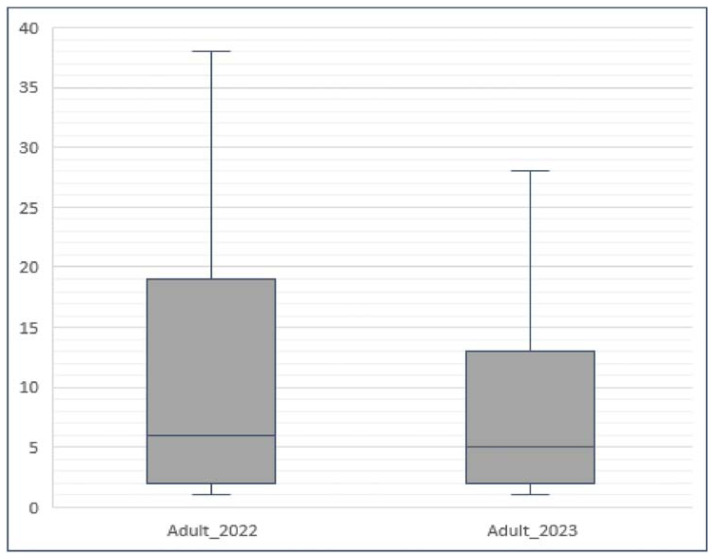
Boxplot of drug consumption in terms of pieces prescribed for adults (40**–**69) years in 2022 and 2023. ( p < 0.001). Adult = 40**–**69 years.

Statistically significant results emerged from the comparison among adults. Findings demonstrated a reduction in the number of prescribed drugs from 2022 to 2023 in this category ( *p* < 0.001), as highlighted in the boxplot depicted in Fig. 6.

**Fig. 7:**
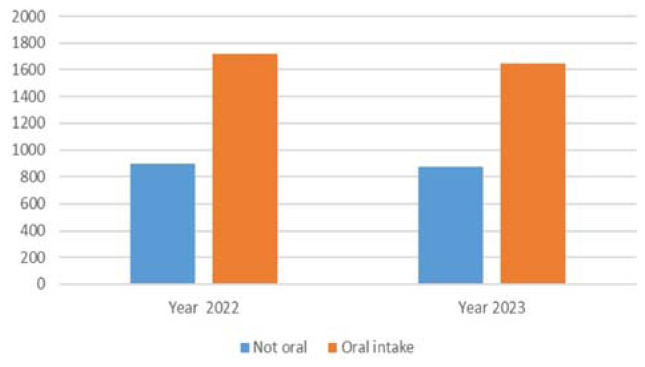
Oral and other routes of administration. Rapid-onset opioids were not considered.

The consumption of opioids, differentiating between oral and other routes of administration, is reported in Fig. 7. Fentanyl citrate was not included in the analysis. In 2022 and 2023, the oral route accounted for 67% and 65.4%, respectively.

The analysis progressed to evaluate patterns of ROOs consumption categorized by age and gender. The analysis showed a higher dosage (>267 mcg) predominance among women, whereas a lower dosage (<133 mcg) was calculated mostly for men (Table 5).

## 4. Discussion

Our analysis has unveiled several crucial facets of opioid use in an Italian region, housing a population of approximately one million residents. The analysis has explored the complexities of prescription patterns, covering the period from January 2022 to October 2023.

Assuming that the majority of diagnoses are related to non-oncological pain of mild to moderate intensity [17], we found a prevalent use of weak opioids. Under other estimates, the higher usage falls on adult subjects [18]. While chronic conditions like low back pain and osteoarthritis, which can typically necessitate opioid usage, predominantly affect adults, this data suggests a cautious prescribing approach for the younger population. In our sample, we found that prescriptions were notably limited for individuals under the age of 20, in both years considered (Fig. 2).

Additional prescribing data align with guidelines and recommendations (Fig. 3). The most commonly prescribed drug in both datasets was the paracetamol/codeine combination, making up 32% in 2022 and 34% in 2023, respectively. When considering weak opioids (codeine and tramadol), the calculated percentages were 44.3% in 2022 and 45% in 2023. Furthermore, we found an effective use of the oral administration route [19]. Within transdermal routes, we have also observed a favorable utilization of buprenorphine. This opioid exhibits advantageous pharmacological properties and a safety profile that could be considered suitable for the management of chronic pain [20]. In Italy, slow-release and low-dosage transdermal formulations of buprenorphine are recommended for use in non-oncological chronic pain [21].

The DDD functions as a globally standardized benchmark, simplifying the comparison of treatment protocols that involve different package sizes and similar therapeutic categories. This metric provides clear advantages over simple “number of units” metrics, as it provides a more comprehensive evaluation of the prescribed medication quantity, irrespective of packaging configuration [22]. The World Health Organization (WHO) suggests employing the ATC classification system and the DDD as the preferred units of measurement for drug utilization research [23]. A correlation between prescriptions and DDD (Figs. 3 and 4), indicates that the prescribed dosages align with globally defined dosage standards for each medication. This provides insights into the adherence of prescriptions to recommended treatment protocols and the quantity of medication prescribed concerning the standardized daily dose. Moreover, we found that from 2022 to 2023, the overall quantity of medication prescribed decreased by approximately 18%.

Remarkably, the trend analysis revealed a notable decrease ( *p* < 0.001) in opioid prescriptions for adults aged 40**–**69 from 2022 to 2023. This observation confirms recent epidemiological data on opioid consumption in Italy, indicating an overall decline in expenditure between 2022 and 2021 [24].

A special issue concerns the use of ROOs. Products containing citrate fentanyl are exclusively indicated for treating breakthrough cancer pain. It is a cancer pain phenomenon that occurs, spontaneously or incident due to a precipitating event such as movements or procedures, in patients with an acceptable control of baseline pain through opioid therapy [25**–**27]. Fentanyl is a synthetic opioid that is particularly responsible for the opioid crisis [28]. Its use, consequently, is closely monitored [11]. In general, we identified a low rate of prescriptions with a decreasing trend (7.7% in 2022 and 4.8% in 2023). Nevertheless, the analysis demonstrated a predominance of higher dosages (>267 mcg) among women, whereas a lower dosage (<133 mcg) was calculated for men. This finding requires further investigation addressing concomitant clinical data and implementing multivariate analyses. Additional investigations are required to evaluate the appropriateness of prescribing these medications based on background cancer pain management and to examine the regimen implemented, including whether they are given in proportional or titrated doses [29].

Taken together, these results confirm a careful evaluation of opioid prescriptions, in Italy and are consistent with our previous observations from a nationwide study [30]. Health policies have implemented diverse preventive strategies to address the misuse or inappropriate prescription of opioids [31]. Furthermore, research programs have been bolstered, and there is widespread recommendation for the use of screening tests to identify potential vulnerabilities to addiction [32]. This circumstance might be attributed to the enactment of Italian Law 38/2010 [33]. This law, a pioneering initiative in Europe and one of the early instances worldwide established regulations for clinical practices related to opioid therapy for individuals experiencing chronic pain. Notably, it streamlines the prescription of opioids when deemed essential, concurrently fostering a culture of awareness among healthcare professionals, and implementing control measures to ensure consistent and appropriate prescriptions [33].

On the other hand, our findings suggest the potential risk of falling into a dangerous phenomenon of undertreatment of oncological pain. According to Lo Bianco et al. [34], Italy stands out as a notable exception to the opioid epidemic, and profound opiophobia can be linked to significant harm and poses a clear ethical dilemma. Therefore, these results emphasize the imperative for a nuanced and balanced approach to opioid prescription, ensuring that patients receive optimal pain management without exposing them to unnecessary risks or compromising their therapeutic outcomes [35].

### 4.1. Study limitations

This research has several limitations. The analysis pertains only to a sample of the national population (approximately 1.7%). Furthermore, even though we analyzed the entire prescription dataset, it is possible that some prescriptions were produced outside the reimbursement pathway of the national health system. However, this occurrence is remote and has a limited impact on the overall sample. Additional limitations concern the use of evaluation metrics for opioid analyses. For example, DDD may not be a faithful indicator of opioid consumption, and recalibration of the DDD for many opioids or reporting opioid utilization in oral morphine equivalent doses is often recommended [36]. The paramount limitation of our analysis is the lack of correlations with diagnoses. Prescriptive appropriateness is a crucial factor in understanding the opioid crisis phenomenon. Additionally, it would have been important to distinguish between prescriptions for oncological pathologies and opioid use for non-oncological conditions. Although will address this gap in a dedicated analysis, in this investigation, we chose to provide a comprehensive overview of the prescribing structure within a specific population. Another limitation is the inability to distinguish between prescriptions for acute and chronic conditions. Moreover, the duration of treatment is a crucial aspect of opioid therapy. To address these issues, it is essential to acquire data related to prescriptions specific to each patient. Consequently, the next step is to analyze additional variables from different sources.

### 4.2. AI-based translational perspectives

Future studies will aim at deepening the proposed investigation by adopting advanced data analysis techniques to enlarge and strengthen the preliminary findings that emerged from this investigation. For example, multivariate analyses and regression models could be implemented to assess the influence of multiple factors on drug usage and investigate the complex relationships among the considered population characteristics and drug consumption for different types of medications. Furthermore, including additional patient-related variables, e.g., information extracted from electronic health records (EHRs), will be crucial for the identification of specific clusters within drug users and for evaluating the risk-benefit ratio for different drug types considered [37]. This approach could enable the development of new tools for monitoring therapy effectiveness [38] and compliance as well as for post-marketing surveillance [39].

From a translational perspective, the adoption of models and tools from the artificial intelligence (AI) domain, such as machine learning (ML), can improve this data-driven analysis, potentially detecting hidden patterns and trends in actual drug utilization. In particular, the application of AI-based predictive analytics models could represent a valuable strategy for forecasting opioid prescribing patterns. This could involve developing algorithms that predict future trends based on historical data, helping healthcare providers and policymakers anticipate changes and implement proactive measures [40]. Furthermore, natural language processing techniques can be employed to extract insights from EHRs, physician notes, and patient feedback, analyzing unstructured data to understand the nuances of opioid prescription decisions, patient experiences, and physician considerations. Significantly, ML algorithms can serve to assess the risk of opioid misuse or addiction based on patient characteristics, medical history, and other relevant factors. This predictive approach could aid healthcare professionals in identifying high-risk patients and tailoring interventions accordingly. In this complex scenario, a fascinating prospect is the potential integration of AI algorithms into clinical decision support systems. Therefore, validated tools can assist healthcare providers in making informed decisions about opioid prescriptions, considering patient-specific factors, co-morbidities, and alternative pain management strategies. The aim is the design of personalized pain management interventions. For this purpose, health technologies are particularly suitable [41]. AI-powered wearable devices, virtual reality therapies, or adaptive treatment plans that dynamically respond to patient feedback, can be implemented to optimize pain relief while minimizing opioid use risks.

Translational medicine can greatly benefit from these technologies. For example, AI can play a significant role in designing and validating translational models of pain and opioid behaviors [42]. As proposed by Bumgarner et al. [43], these methodologies hold critical importance in advancing the development of safer non-opioid analgesics and alternative treatment modalities for opioid use disorders. Furthermore, these approaches play a crucial role in the development of novel compounds [44].

Nevertheless, the short-term landscape presents a multitude of challenges to overcome. The efficacy of AI across its various domains heavily relies on the quality of the data it is fed. The well-known maxim ‘garbage in, garbage out’ underscores the critical importance of ensuring that the input data is accurate, reliable, and of high quality. It emphasizes the need for meticulous data curation and quality control processes. The “5 V paradigm” including Volume, Velocity, Variety, Veracity, and Value, refers to the characteristics or dimensions of big data, and it has become a common framework for understanding the challenges and opportunities associated with large and complex datasets [45]. Reinforcing the significance of maintaining rigorous standards in data acquisition and preprocessing is mandatory to unlock the full potential of AI technologies [46]. Finally, ethical considerations in AI-driven healthcare are a key issue [47]. Research, scientific societies, and well-calibrated regulatory processes will be crucial for exploring matters associated with algorithmic bias, concerns about patient privacy, and the accountability of healthcare professionals in integrating AI recommendations into their decision-making workflows [48].

### 4.3. Conclusion

Despite the several limitations, particularly concerning the motivation for the prescription, which call for a judicious interpretation of the findings, our investigative efforts have brought to light careful opioid prescription in an Italian population. These findings align with and reinforce the earlier emphasis on the varied impacts of the opioid crisis on a global scale. Finally, results reaffirm the importance of nuanced considerations in prescribing practices, recognizing the distinct contexts within which opioid utilization occurs and the necessity for tailored approaches in addressing the complexities of pain management.

## Figures and Tables

**Table 1:** Cumulative use of opioids in 2022 and 2023.

Year 2022. Medication	Cumulative use^	Year 2023. Medication	Cumulative use
**BUPRENORPHINE** *	**8096**	**BUPRENORPHINE** *	**8056**
00**–**09	8	00**–**09	16
10**–**19	1	10**–**19	2
20**–**29	4	20**–**29	7
30**–**39	52	30**–**39	39
40**–**49	274	40**–**49	320
50**–**59	705	50**–**59	695
60**–**69	1528	60**–**69	1524
70**–**79	2491	70**–**79	2530
80+	3033	80+	2923
**BUPRENORPHINE HYDROCHLORIDE**	**2**	**FENTANYL**	**12,128**
60**–**69	1	00**–**09	19
70**–**79	1	10**–**19	1
**FENTANYL**	**15,337**	20**–**29	14
00**–**09	14	30**–**39	162
10**–**19	34	40**–**49	712
20**–**29	110	50**–**59	2219
30**–**39	373	60**–**69	2727
40**–**49	893	70**–**79	3618
50**–**59	2492	80+	2656
60**–**69	3547	**FENTANYL CITRATE** ○	**5804**
70**–**79	4492	00**–**09	1
80+	3382	10**–**19	10
**FENTANYL CITRATE** ○	**12,107**	20**–**29	21
00**–**09	3	30**–**39	399
10**–**19	7	40**–**49	1295
20**–**29	148	50**–**59	949
30**–**39	1213	60**–**69	1369
40**–**49	3951	70**–**79	1211
50**–**59	2328	80+	549
60**–**69	1949	**IBUPROFEN/CODEINE PHOSPHATE HEMIHYDRATE** ^‡^	**1540**
70**–**79	1835	00**–**09	1
80+	673	10**–**19	12
**IBUPROFEN/CODEINE PHOSPHATE HEMIHYDRATE**	**2104**	20**–**29	19
00**–**09	**–**1	30**–**39	60
10**–**19	3	40**–**49	169
20**–**29	32	50**–**59	298
30**–**39	92	60**–**69	343
40**–**49	196	70**–**79	378
50**–**59	362	80+	260
60**–**69	504	**HYDROMORPHONE HYDROCHLORIDE**	**11**
70**–**79	569	50**–**59	7
80+	345	60**–**69	1
**HYDROMORPHONE HYDROCHLORIDE**	**143**	70**–**79	3
30**–**39	2	**MORPHINE HYDROCHLORIDE**	**107**
50**–**59	11	40**–**49	2
60**–**69	9	50**–**59	24
70**–**79	95	60**–**69	49
80+	26	70**–**79	14
**MORPHINE HYDROCHLORIDE**	**756**	80+	18
20**–**29	418	**MORPHINE HYDROCHLORIDE TRIHYDRATE**	**1746**
30**–**39	2	00**–**09	1
40**–**49	43	20**–**29	122
50**–**59	154	30**–**39	366
60**–**69	83	40**–**49	26
70**–**79	35	50**–**59	538
80+	21	60**–**69	417
**MORPHINE HYDROCHLORIDE TRIHYDRATE**	**881**	70**–**79	169
20**–**29	164	80+	107
30**–**39	17	**MORPHINE SULFATE**	**744**
40–49	111	00**–**09	1
50**–**59	156	20**–**29	3
60**–**69	161	30**–**39	17
70**–**79	194	40**–**49	67
80+	78	50**–**59	125
**MORPHINE SULFATE**	**1412**	60**–**69	249
00**–**09	2	70**–**79	178
10**–**19	6	80+	104
30**–**39	23	**OXYCODONE HYDROCHLORIDE**	**1381**
40**–**49	110	00**–**09	4
50**–**59	357	20**–**29	8
60**–**69	344	40**–**49	500
70**–**79	428	50**–**59	179
80+	142	60**–**69	373
**OXYCODONE HYDROCHLORIDE**	**1670**	70**–**79	177
10**–**19	23	80+	140
20**–**29	26	**OXYCODONE HYDROCHLORIDE/NALOXONE**	**11,478**
30**–**39	48	00**–**09	21
40**–**49	277	10**–**19	12
50**–**59	333	20**–**29	63
60**–**69	467	30**–**39	139
70**–**79	393	40**–**49	666
80+	103	50**–**59	1639
**OXYCODONE HYDROCHLORIDE/NALOXONE**	**15,341**	60**–**69	2621
00**–**09	20	70**–**79	3326
10**–**19	8	80+	2991
20**–**29	60	**OXYCODONE HYDROCHLORIDE/PARACETAMOL**	**7592**
30**–**39	290	00**–**09	11
40**–**49	937	10**–**19	7
50**–**59	2027	20**–**29	22
60**–**69	3578	30**–**39	311
70**–**79	4297	40**–**49	464
80+	4124	50**–**59	1229
**OXYCODONE HYDROCHLORIDE/PARACETAMOL**	**8384**	60**–**69	1699
00**–**09	5	70**–**79	2322
10**–**19	5	80+	1527
20**–**29	38	**PARACETAMOL/CODEINE PHOSPHATE**	**42,267**
30**–**39	245	00**–**09	78
40**–**49	597	10**–**19	80
50**–**59	1403	20**–**29	374
60**–**69	1851	30**–**39	805
70**–**79	2406	40**–**49	2168
80+	1834	50**–**59	5362
**PARACETAMOL/CODEINE PHOSPHATE**	**51,381**	60**–**69	7977
00**–**09	40	70**–**79	13,349
10**–**19	108	80+	12,074
20**–**29	452	**TAPENTADOL**	**16,836**
30**–**39	1066	00–09	39
40**–**49	2957	10–19	3
50**–**59	6491	20**–**29	72
60**–**69	10,165	30**–**39	168
70**–**79	16,209	40**–**49	872
80+	13,893	50**–**59	2681
**TAPENTADOL HYDROCHLORIDE**	**21,258**	60**–**69	3478
00**–**09	8	70**–**79	5162
10**–**19	5	80+	4369
20**–**29	83	**TRAMADOL HYDROCHLORIDE**	**11,435**
30**–**39	265	00**–**09	33
40**–**49	1077	10**–**19	9
50**–**59	3277	20**–**29	75
60**–**69	4281	30**–**39	314
70**–**79	6826	40**–**49	722
80+	5436	50**–**59	2375
**TRAMADOL HYDROCHLORIDE**	**18,521**	60**–**69	2871
00**–**09	28	70**–**79	2788
10**–**19	27	80+	2248
20**–**29	140	**TRAMADOL HYDROCHLORIDE/DEXKETOPROFE**	**29**
30**–**39	565	30**–**39	1
40**–**49	1501	40**–**49	6
50**–**59	3429	50**–**59	18
60**–**69	4428	60**–**69	4
70**–**79	4567	**TRAMADOL HYDROCHLORIDE/DEXKETOPROFE**	**3**
80+	3836	50**–**59	3
**TRAMADOL HYDROCHLORIDE/DEXKETOPROFEN**	**48**	**TRAMADOL HYDROCHLORIDE/PARACETAMOL**	**7**
30**–**39	3	30**–**39	1
40**–**49	8	40**–**49	1
50**–**59	27	50**–**59	3
60**–**69	10	60**–**69	2
**TRAMADOL HYDROCHLORIDE/PARACETAMOL**	**2**		

^ The cumulative use refers to the number of prescriptions and drug packages for each medication.

* Buprenorphine patch.

○ Rapid-onset opioids.

† In Italy, codeine is marketed exclusively in combination.

**Table 2:** Pairwise comparisons of age groups 2022 (Dunn**–**Bonferroni test).

Sample1–Sample2^	Significance	Adjusted significance
Elderly_2022 - Young_2022	0.435	1.000
Elderly_2022 - Adult_2022	**0.002**	**0.005**
Young_2022 - Adult_2022	0.215	0.645

^ Each row tests the null hypothesis that Sample 1 and Sample 2 are the same. Asymptotic significances (2-sided tests) are displayed. The significance level is *p* < 0.050 (in bold).

○ Significance values have been adjusted by the Bonferroni correction for multiple tests. Young <39 years; Adult = 40**–**69; Elderly >70.

**Table 3:** Pairwise comparisons of age groups 2023 (Dunn**–**Bonferroni test).

Sample1–Sample2^	Significance	Adjusted significance
Young_2023 **–** Elderly_2023	0.337	1.000
Young_2023 - Adult_2023	0.103	0.308
Elderly_2023 - Adult_2023	0.190	0.570

^ Each row tests the null hypothesis that Sample 1 and Sample 2 are the same. Asymptotic significances (2-sided tests) are displayed. The significance level is *p* < 0.050.

○ Significance values have been adjusted by the Bonferroni correction for multiple tests. Young <39 years; Adult = 40**–**69; Elderly >70.

**Table 4:** Differences in opioid prescriptions between 2022 and 2023.

Year 2022	Year 2023	*p*-value
**Young**	Young	0.125
**Adult**	Adult	0.013
**Old**	Old	0.674

Young <39 years; Adult = 40**–**69; Elderly >70.

**Table 5:** Rapid onset opioid consumption.

Year 2022. Dosages^	Cumulative use	Year 2023. Dosages	Cumulative use
**High dosage**	**6297**	**High dosage**	**153**
** 00–09**	**2**	** 00–09**	**1**
F	2	M	1
** 10–19**	**18**	** 20–29**	**6**
F	18	F	1
** 20–29**	**18**	M	5
F	18	** 30–39**	**7**
** 30–39**	**200**	F	1
F	200	M	6
** 40–49**	**3668**	** 40–49**	**17**
F	3668	M	8
** 50–59**	**765**	F	9
F	765	** 50–59**	**32**
** 60–69**	**897**	F	10
M	897	M	22
** 70–79**	**670**	** 60–69**	**44**
M	670	F	18
** 80**+	**59**	M	26
M	59	** 70–79**	**31**
**Low dosage**	**4526**	F	15
** 00–09**	**1**	M	16
F	1	** 80**+	**15**
** 20–29**	**37**	M	6
F	37	F	9
** 30–39**	**131**	**Low dosage**	**168**
F	131	** 10–19**	**2**
** 40–49**	**1338**	F	2
F	1338	** 20–29**	**1**
** 50–59**	**162**	M	1
F	162	** 30–39**	**7**
** 60–69**	**2427**	M	3
M	2427	F	4
** 70–79**	**430**	** 40–49**	**20**
M	430	M	9
**Medium dosage**	**615**	F	11
** 10–19**	**40**	** 50–59**	**31**
F	40	M	15
** 20–29**	**3**	F	16
F	3	** 60–69**	**34**
** 30–39**	**53**	F	14
F	53	M	20
** 40–49**	**127**	** 70–79**	**36**
F	127	F	18
** 50–59**	**169**	M	18
F	35	** 80**+	**37**
M	134	M	18
** 60–69**	**148**	F	19
M	148	**Medium dosage**	**110**
** 70–79**	**75**	** 20–29**	**1**
M	75	M	1
		** 30–39**	**5**
		M	2
		F	3
		** 40–49**	**10**
		M	3
		F	7
		** 50–59**	**21**
		M	10
		F	11
		** 60–69**	**25**
		M	12
		F	13
		** 70–79**	**29**
		M	14
		F	15
		** 80**+	**19**
		F	9
		M	10

^ To categorize the diverse formulations of ROO, we segmented the dosages into three tiers: low (<133 mcg), medium (133**–**267 mcg), and high dosages (>267 mcg).
